# Toluene associated schizophrenia-like psychosis

**DOI:** 10.4103/0019-5545.58307

**Published:** 2009

**Authors:** Naren P. Rao, Arun Gupta, K. Sreejayan, Prabhat K. Chand, Vivek Benegal, Pratima Murthy

**Affiliations:** Department of Psychiatry, National Institute of Mental Health and Neurosciences, Bangalore - 560 029, India. E-mail: docnaren@gmail.com

Sir,

Inhalants are commonly abused drugs in adolescent population though often unrecognized. On long-term use they can result in serious sequels like optic atrophy and psychosis, resembling schizophrenia, and thus need awareness among physicians and psychiatrists. While there have been case reports on inhalant abuse in India,[1] there has been no report describing psychosis following toluene use. In this report we describe a patient presenting with psychosis following toluene use and improvement of psychosis with abstinence.

Mr. A., a 23-year-old man, was brought to the hospital in 2006, with a history of huffing correction fluid (eraz-ex), containing toluene since eight years. He was using around 300 ml per day. He had craving, tolerance, and continued using it despite the harm. Since three years the family reported behavioral changes in him, in the form of suspiciousness that neighbors were trying to harm him, making gestures with sexual connotations, and hearing voices commenting on his actions. He would hoard garbage and dead crows. These behaviors were persistent and worsened with time, without features suggestive of altered sensorium. He was never abstinent of the inhalant. He also complained of blurred vision since two years. There was no past or family history of any psychotic episode. He did not report abuse of any other drug except nicotine.

On examination he had delusions of persecution, reference, a bizarre delusion that a five-headed snake was inside him, and auditory hallucinations. He scored 50 on the Brief Psychiatric Rating Scale (BPRS) on the day of admission. Physical examination showed hyperemia of fundus and blurred vision in both eyes. His breath smelt of toluene. There were no other neurological abnormalities on physical examination. A drug screen was negative for cannabis, opioid, and benzodiazepines. A CT scan of the brain was normal. Mr. A was diagnosed as having volatile solvent dependence, solvent-induced, schizophrenia-like psychotic disorder (ICD-10). The ophthalmologist's opinion was taken and he was diagnosed with toxic maculopathy.

He was treated with diazepam for sleep disturbance and agitation. He was abstinent of all substances during hospital stay. Within two weeks all his psychotic symptoms subsided without any antipsychotic. He scored 33 on the tenth day and 28 on the fifteenth day on BPRS. However, his visual impairment remained unchanged.

The index patient presented with chronic solvent abuse and persistent bizarre delusions and hallucinations and the symptoms decreased within 15 days following abstinence from toluene, without antipsychotic medication. Hence we attribute the symptoms to chronic toluene use. Optic neuropathy has been described with toluene abuse,[2] as seen in the index patient. There has been a report of chronic industrial exposure to toluene resulting in psychotic symptoms.[3] Treatment of inhalant-induced psychotic disorder has primarily involved the use of antipsychotics and anticonvulsants such as carbamazepine and haloperidol.[4] However, few workers report that central nervous system damage is repaired with abstinence and time alone.[5] In the index patient, the psychotic symptoms subsided with abstinence and supportive treatment, without requiring treatment with antipsychotics supporting the view mentioned earlier. However, more studies need to be conducted, to confirm the association of solvents and psychosis.

## References

[1] Basu D, Jhirwal OP, Singh J, Kumar S, Mattoo SK (2004). Inhalant abuse by adolescents: A new challenge for Indian physicians. Indian J Med Sci.

[2] Keane JR (1978). Toluene optic neuropathy. Ann Neurol.

[3] Goldbloom D, Chouinard G (1985). Schizophreniform psychosis associated with chronic industrial toluene exposure: Case report. J Clin Psychiatry.

[4] Hernandez-Avila CA, Ortega-Soto HA, Jasso A, Hasfura-Buenaga CA, Kranzler HR (1998). Treatment of inhalant-induced psychotic disorder with carbamazepine versus haloperidol. Psychiatr Serv.

[5] Westermeyer J (1987). The psychiatrist and solvent-inhalant abuse: Recognition, assessment, and treatment. Am J Psychiatry.

